# The Cytolethal Distending Toxin Effects on Mammalian Cells: A DNA Damage Perspective

**DOI:** 10.3390/cells3020592

**Published:** 2014-06-11

**Authors:** Elisabeth Bezine, Julien Vignard, Gladys Mirey

**Affiliations:** 1 INRA, UMR1331, Toxalim, Research Centre in Food Toxicology, F-31027 Toulouse, France; E-Mail: elisabeth.bezine@toulouse.inra.fr; 2 Genotoxicity Signaling Team, Toxalim INRA, 180 Chemin de Tournefeuille BP 93173, F-31027 Toulouse, France; 3 Université de Toulouse, INP, UMR1331, Toxalim, F-31000 Toulouse, France; 4 Université de Toulouse, UPS, UMR1331, Toxalim, F-31062 Toulouse, France

**Keywords:** Gram-negative bacteria, cytolethal distending toxin, DNA damage response, double-strand breaks, cell cycle checkpoints, replicative stress

## Abstract

The cytolethal distending toxin (CDT) is produced by many pathogenic Gram-negative bacteria and is considered as a virulence factor. In human cells, CDT exposure leads to a unique cytotoxicity associated with a characteristic cell distension and induces a cell cycle arrest dependent on the DNA damage response (DDR) triggered by DNA double-strand breaks (DSBs). CDT has thus been classified as a cyclomodulin and a genotoxin. Whereas unrepaired damage can lead to cell death, effective, but improper repair may be detrimental. Indeed, improper repair of DNA damage may allow cells to resume the cell cycle and induce genetic instability, a hallmark in cancer. *In vivo*, CDT has been shown to induce the development of dysplastic nodules and to lead to genetic instability, defining CDT as a potential carcinogen. It is therefore important to characterize the outcome of the CDT-induced DNA damage and the consequences for intoxicated cells and organisms. Here, we review the latest results regarding the host cell response to CDT intoxication and focus on DNA damage characteristics, cell cycle modulation and cell outcomes.

## 1. Introduction

The cytolethal distending toxin (CDT) was discovered and described as a heat-labile exotoxin, from both *Escherichia coli* (EcCDT) and *Campylobacter jejuni* (CjCDT), able to induce the distension and death of eukaryotic cells [1,2]. Later, different bacterial strains obtained from human clinical isolates were shown to produce CDT, including *Haemophilus ducreyi* (HdCDT) [3], *Aggregatibacter actinomycetemcomitans* (AaCDT) [4] and enterohepatic *Helicobacter cinaedi* [5], all being Gram-negative pathogenic bacterial strains. In addition, *Salmonella enterica* serovar typhi (*S. typhi*) displays a CDT-like activity without expressing a typical CDT toxin [6]. Finally, CDT is also found in bacteria colonizing animals, such as *Helicobacter* species in poultry [7], mice and woodchuck [8] (reviewed in [9]). Up to now, no Gram-positive CDT producing bacteria have been characterized.

Globally, eukaryotic cell exposure to CDT leads to a characteristic cytotoxicity associated with a cell distension phenomenon. CDT also induces a cell cycle arrest dependent on the DNA damage response (DDR), triggered by DNA double-strand breaks (DSBs). In addition to CDTs, only a few bacterial genotoxins have been described, among them the *E. coli* Usp (uropathogenic-specific protein) [10] and colibactin, characterized in extra-intestinal commensal and pathogenic *E. coli* strains [11]. Regarding the pathological significance, *E. coli* Usp is associated with urinary tract infection [12], whereas colibactin has been shown to promote colorectal cancer [13]. In this review, we will focus on CDT and briefly present the structural features of CDT and the trafficking of the catalytic moiety to the host cell nucleus. We will then describe the host cell response to CDT intoxication and, finally, discuss the CDT-related DNA damage characteristics.

### 1.1. CDT-Related Pathogenicity

The CDT toxin has been involved in diseases development and is thus considered as a virulence factor [14,15]. For example, the pathophysiologic role of CDT has been clearly shown in a rat model for *C. jejuni*, where only a catalytically-active CjCDT induced damage to the epithelial barrier, diarrhea and severe inflammation of the entire gastro-intestinal tract [16,17]. CDT has also been implicated in *Helicobacter hepaticus* pathogenicity, as the toxin is key in the development of hepatic dysplastic nodules in an immunocompetent mouse model [18]. Finally, in *S. typhi*, the pathogen responsible for typhoid fever causing more than 200,000 deaths worldwide per year, the role of the CDT-like typhoid toxin has been characterized. In contrast to a catalytic mutant of the toxin (a mutant of the CDT catalytic moiety), the systemic administration of the purified wild-type typhoid toxin in a mouse model induces almost all of the typhoid fever symptoms [19]. Taken together, these data clearly show that the role of CDT in different pathogenic contexts mainly relies on the catalytic activity of the toxin.

### 1.2. CDT is a Tripartite A-B Exotoxin

The CDT holotoxin is made of three subunits, CdtA, CdtB and CdtC, encoded by three genes organized in one operon [20,21]. The structures of AaCDT and of HdCDT have been determined [22,23], showing that CDT displays an A-B architecture, like many other exotoxins, where CdtB is the catalytic A-subunit. The B-moiety, essential for the holotoxin binding to the host cell membrane, is composed of the CdtA and CdtC subunits. CDT can therefore be classified as an A-B_2_ exotoxin. To date, the only known exception is the typhoid toxin of *S. typhi*, in which the *CdtB* gene is not associated with *CdtA* and *CdtC*, but to *PltA* and *PltB* [24], encoding, respectively, the pertussis-like toxin A (homologous to the pertussis toxin ADP-ribosyltransferase subunit) and the pertussis-like toxin B (homologous to one of the pertussis B subunits) [25]. The structure of the typhoid toxin has been solved [19] and shown to be an A_2_–B_5_ toxin, the B5 regulatory subunit being composed of a pentameric PltB, whereas the A2 catalytic subunit is composed of the StCdtB and PltA proteins, covalently linked by a disulfide bond.

**Figure 1:**
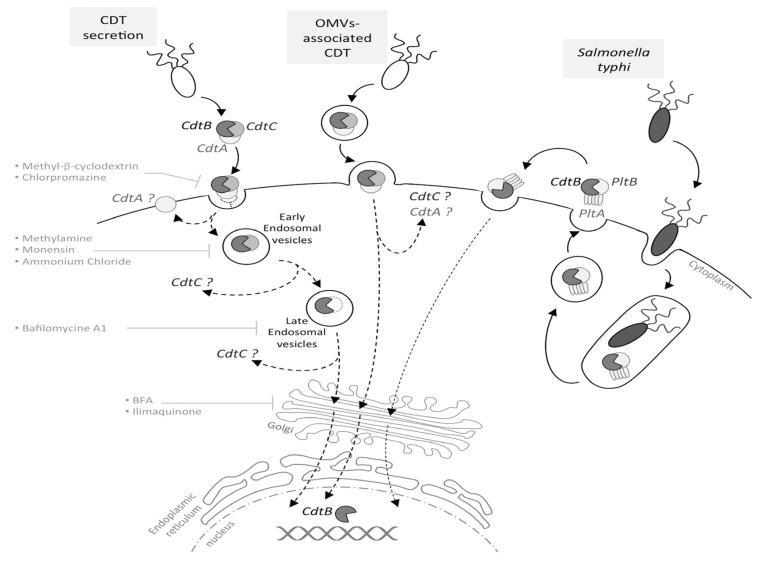
Cytolethal distending toxin (CDT) internalization and trafficking. Depending on the bacteria, CDT may be secreted freely, into outer membrane vesicles (OMVs) or, in the particular case of *Salmonella typhi*, into intracellular vesicles. In the case of a CDT extracellular secretion, CdtA and CdtC are involved in the toxin binding to the eukaryotic membrane. Once bound, CdtA remains associated with the membrane, while CdtC and CdtB are internalized, with CdtB being relocated to the nucleus by a retrograde transport pathway, via early and late endosomes. This has been demonstrated using inhibitors, such as methyl-β-cyclodextrin, methylamine, bafilomycin A1, BFA, *etc.* For OMV-secreted CDT, the toxin is internalized into the host cell through the OMV fusion with the eukaryotic membrane. CdtB is relocated to the nucleus by an undescribed pathway (dotted arrow), and the CdtA and CdtC outcome in the cytoplasm is still unknown. The typhoid toxin production requires *S. typhi* internalization into the host cell; thereafter, the toxin must be secreted to be active. The typhoid toxin interacts with the eukaryotic membrane and is endocytosed, and CdtB is relocated to the nucleus.

CdtA, CdtB and CdtC present a signal sequence and are, therefore, directed to the general secretory pathway, leading to CDT secretion [26,27]. However, CDT may also be released through outer membrane vesicles (OMVs), fusing with the host plasma membrane via lipid rafts [28,29,30]. The *S. typhi* toxin is once again an exception, as infection studies revealed that the bacterial uptake into host cells triggers the CdtB/PltA/PltB expression, leading to the formation of an intracellular multipartite toxin. Following its production, the typhoid toxin is secreted into the extracellular environment and then interacts, in an autocrine and paracrine way, with the eukaryotic plasma membrane to be internalized and to exert its cytotoxic activity [24], as the inhibition of the typhoid toxin export in the extracellular medium inhibits its effects (Figure 1).

### 1.3. From CDT Host Cell Binding to CdtB Nuclear Localization

CDT toxins are produced by bacterial strains located in different niches, implying that all CDTs are not secreted in the same microenvironment (different epithelia types, presence of mucus or not, *etc.*), and this raises questions regarding the cell specificity of the CDT toxins [31]. The CdtB subunit is the most conserved of the subunits among all CDT-producing bacteria strains [32]. On the other hand, significant sequence variability is found between CdtC and CdtA homologs. As CdtA and CdtC subunits are essential to the CDT binding, with CdtB alone not being able to bind at the host cell surface [33,34], some authors hypothesized that this variability may allow CDT to interact specifically with different cell types of the host niches [35]. However, if CdtA and CdtC are involved in the CDT binding to host cells, the nature of the CDT receptor only begins to emerge. Recently, genetic screens identified several eukaryotic cell candidates important for CDT intoxication [36,37]. If different CDT toxins share some host factors as the SGMS1 gene encoding the sphingomyelin synthase 1, some specific host proteins were also found. For example, EcCDT-I specifically requires a putative G protein-coupled receptor, TMEM181, shown to co-immunoprecipitate with CDT [36]. The CDT receptor(s) identification may ultimately provide insights regarding cellular tropism and shed light on host-bacteria interactions.

CDT internalization in host cells seems to occur through receptor-mediated endocytosis [38], where the host receptor bound by the toxin can be considered as a cargo hijacked from its normal cellular function. However, this does not rule out the possibility that CDT enters through other endocytic pathways, such as caveolae, given the importance of cholesterol-rich domains [39,40,41,42], or through cholesterol-independent endocytic pathways [43].

Recently, AaCdtA was shown to stay at the cell surface, whereas AaCdtC was found both on the host cell surface and in the cytosol, AaCdtB being localized at the ER and later at the nucleus [43]. CdtB relocation to the nucleus was also observed after CdtB microinjection into eukaryotic cell cytoplasm [44,45]. Four hours after microinjection, CjCdtB and AaCdtB are located into the nucleus, demonstrating that the nuclear localization of CdtB from the cytoplasm to the nucleus does not require CdtA and CdtC subunits.

After cell entry by endocytosis, HdCdtB seems to follow a retrograde endosome-Golgi traffic to the endoplasmic reticulum (ER) [38], however without requiring the ER-associated degradation (ERAD) pathway usually exploited by ER-translocating toxins [39]. HdCdtB may be directly translocated from the ER to the nucleus, as it could not be seen freely in the intoxicated cells cytosol [46]. Interestingly, the endosomal disruption stops the HdCDT transport, but has no effect on EcCDT-III [47], supporting the hypothesis that different CDT toxins may use different trafficking pathways, possibly through different cargo interactions. Important domains for CdtB nuclear transport have been characterized with green fluorescent protein (GFP) fusions. In the CdtB N-terminal region, an 11-amino acid peptide was found, essential for both nuclear localization and toxin-induced cellular effects [45]. This sequence could be replaced by the nuclear localization signal (NLS) of the SV40 large T-antigen [45], suggesting that CdtB nuclear localization is crucial for CDT cytotoxicity. In addition, two potential NLSs have been identified in the C-terminal part of EcCdtB-II [48], whose deletion produces differential CdtB localizations, suggesting specific functions for these NLS sequences.

To summarize, the CDT trafficking current model involves a stepwise holotoxin disassembly process with: (1) CdtA subunit retention at the plasma membrane, after the CDT toxin binds to a yet unidentified receptor; (2) endocytosis of the CdtB-CdtC complex; and (3) CdtB nuclear localization by a retrograde transport pathway (Figure 1). In addition, CdtC could inhibit the CdtB catalytic activity, as suggested by structural data showing that the N-terminus tail of CdtC occludes the CdtB active site [22]. Hence, once it has entered into the host cell and during its intracellular trafficking, CdtB seems locked-in and has to be released from the CdtC interaction to be active.

## 2. DNA Damage-Related Cellular Outcomes of CDT Intoxication

Since the discovery of CDT, the cellular response to CDT intoxication has been better and better characterized. CDT induces a cell cycle arrest (at the G2/M transition and, depending on the cell type, at the G1/S transition), accompanied by a cellular distention and, eventually, cell death [49,50,51,52]. These CDT effects showed similarities with those exerted by some DNA damaging agents, such as ionizing radiation (IR) and etoposide, activating similar pathways [50,52,53]. In light of this observation, the apparent sequence homology between CdtB and the endonuclease/exonuclease/phosphatase family encouraged researchers to compare more precisely, among these proteins, CdtB with a well-known nuclease: deoxyribonuclease I (DNase I) [44,54]. As most of the DNase I residues essential for enzymatic activity are strikingly conserved in the different CdtB homologues, potential sites involved in the CdtB nuclease activity have been determined. The corresponding mutants failed to induce any distension, cell death or cell cycle arrest, showing that the CdtB catalytic activity is responsible for the observed cellular effects and that CdtB has a functional homology with DNase I [44,54]. Finally, the CdtB nuclease activity has been demonstrated *in vitro* by incubating a super-coiled plasmid DNA with the whole CDT holotoxin or with CdtB alone (see below). We reintroduce here the important concepts to study the DNA damage response pathway activation and relate them with the observations made after CDT treatment.

### 2.1. The CDT-Activated DNA Damage Response

In order to replicate correctly and to maintain the stability of their genetic information, eukaryotic cells display systems for controlling the genome integrity. Cells continuously undergo DNA damage generated by different sources (environment, metabolism, *etc.*). To survive in the presence of DNA lesions, the cell has developed mechanisms to detect and signal the damage. These interconnected pathways are referred to as the DNA damage response (DDR).

#### 2.1.1. Introduction to the DDR

The DNA damage-related activation of checkpoints is divided into three stages: (i) the recognition of DNA damage by sensor proteins (MRN and Ku complexes, RPA), which rapidly activate specific phosphatidylinositol 3-kinase-related protein kinases (ATM, ATR, DNA-PK) [55]; (ii) the signal amplification by transducing proteins (CHK1, CHK2); which (iii) activates an appropriate cellular response by effectors proteins (p53, CDC25, *etc.*). This cell response initiates the cell cycle regulation, the activation of DNA repair pathways and, in some cases, cell death pathways [56].

**Figure 2:**
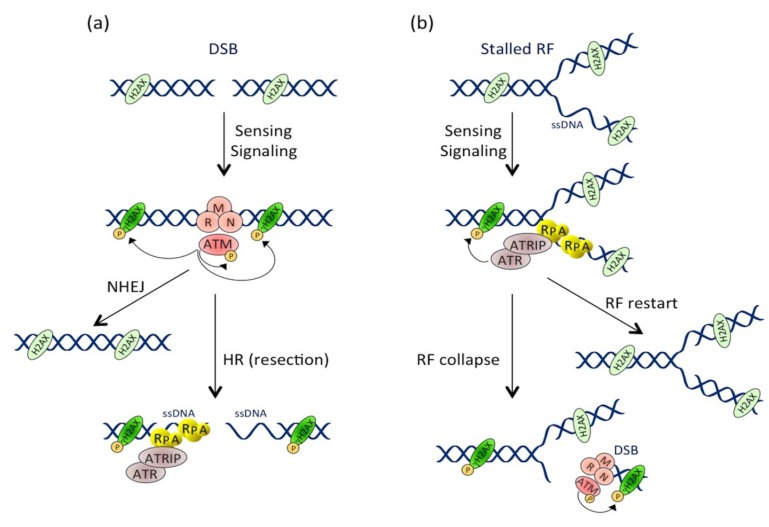
Activation and crosstalk between the ATM and ATR pathways. (**a**) Pathway activation at the double-stranded break (DSB). DSB formation induces the MRN-dependent ATM recruitment. ATM then phosphorylates numerous substrates, including itself and H2AX near the DSB site. DSB repair occurs through non-homologous end joining (NHEJ) or homologous recombination (HR) mechanisms (see Section 3.2 for details). During HR, resection of the DSB extremities produces ssDNA stretches that are coated by RPA, leading to ATRIP-dependent ATR recruitment and activation. (**b**) Pathway activation during replicative stress. Replication fork (RF) stalling induces ssDNA formation that is rapidly coated by RPA. This structure is recognized by ATRIP, which drives the recruitment and activation of ATR. Besides phosphorylating its other substrates, ATR locally phosphorylates H2AX. RF can restart in case of transient stress or collapse after prolonged stresses. RF collapse induces DSB formation and ATM pathway activation, leading to a second and more important wave of H2AX phosphorylation.

Two key signaling pathways are activated in response to DNA damage: the ATM-CHK2 and the ATR-CHK1 pathways. The ATM-CHK2 pathway is activated with the response to DSB-inducing agents. Following the generation of a DSB, the MRN complex, consisting of MRE11, RAD50 and NBS1, recruits the ATM kinase to the site of injury [57]. Once at the DSB site, ATM is activated by autophosphorylation and phosphorylates hundreds of substrates, including CHK2 and p53. Meanwhile, ATM phosphorylates the H2AX histone (then called γH2AX), several megabases around the DSB site [58], allowing signal amplification. The activated CHK2 phosphorylates various substrates, including p53 and the CDC25 phosphatases family. By contrast, the ATR-CHK1 pathway is activated by the accumulation of single-stranded DNA (ssDNA), particularly during the stalling of replication forks (RFs). Indeed, when replication is blocked by DNA lesions (SSB, DSB, inter-strand and intra-strand crosslinks, base modifications or adducts), DNA polymerase is uncoupled from the replicative helicase, which continues to unwind the DNA and, thus, generates ssDNA. ssDNA is recognized by the RPA protein complex, which protects and stabilizes it, and the accumulation of RPA-coated ssDNA at stalled RFs induces the recruitment of the ATR/ATRIP complex [59]. ATR then phosphorylates the CHK1 transducing protein [60]. ATR can, like ATM, phosphorylate H2AX [61], p53 and many cell cycle regulators (such as CDC25A, CDC25C and Wee1). In conclusion, whatever the activated pathway, the ATR-CHK1 or ATM-CHK2 activation will lead to major protein phosphorylation, involved in various cellular processes, including cell cycle regulation, DNA repair and programmed cell death [62]. However, it has to be underlined that many crosstalk exist between the ATM and ATR pathways (reviewed in [63]). Indeed, although CHK2 is the ATM primary target, ATM can also phosphorylate CHK1. In addition, processing of DSBs during the homologous recombination pathway (HR) generates stretches of ssDNA, leading to the ATR pathway activation [64]. Conversely, prolonged replicative stress can provoke RF collapse [65], resulting in DSB formation and ATM-CHK2 pathway activation (Figure 2). In summary, according to the type of DNA injury, the ATM and ATR pathways can be specifically or sequentially activated, resulting in the cell cycle arrest, the activation of the DNA repair machinery and, potentially, in cell death.

#### 2.1.2. CDT Activates the DNA Damage Response

**Figure 3:**
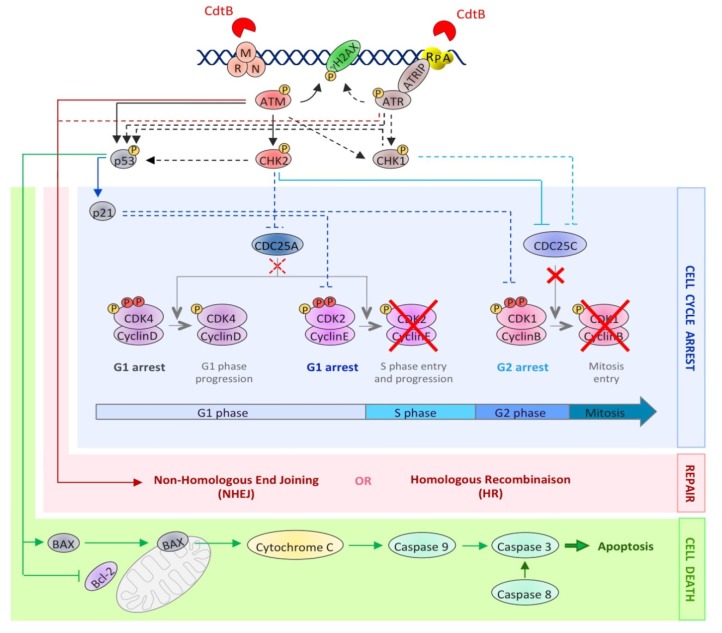
The activation of the DNA damage response upon CDT exposure. This picture depicts the DDR molecular events induced after CDT intoxication. The dotted lines represent well-studied events occurring during the DDR, but not yet demonstrated in the context of CDT exposure. CdtB-induced DNA lesions are detected by sensors, such as the MRN complex and RPA, resulting in the recruitment and the activation of the PI3K related kinases, ATM and ATR. ATM and ATR then phosphorylate hundreds of substrates, including H2AX, CHK1, CHK2 and p53 (black arrows). This signaling cascade results in the regulation of cell cycle modulators (blue lines), through the inhibition of CDC25C phosphatase by CHK2 and, possibly, CHK1. Phosphorylated CDC25C is unable to activate the cyclin B/CDK1 complex (red crosses), essential for mitotic entry. Moreover, the p53-dependent accumulation of p21 blocks cells in G1 by inhibiting the CDK2/cyclin E complex. At the same time, the DSB repair mechanisms (NHEJ and HR) are activated by ATM and ATR (red arrows). However, if the level of DNA lesions is too severe, the process of cell death is initiated (green arrows). Apoptotic cell death can be induced by an intrinsic pathway involving p53 activation, leading to an increase in the BAX level, the sequestration and inactivation of Bcl-2, the mitochondrial release of cytochrome C and caspase 9 activation. Apoptosis can also be induced through the activation of the extrinsic pathway, involving caspase 8 activation. In both cases, this leads to caspase 3 activation and apoptotic cell death.

Many studies have compared the cellular effects of CdtB with DSB induced by IR. The first report deals with the cell cycle arrest induced in response to HdCDT or IR, in HL(human embryonic lung)-fibroblasts and HEp-2 cells [66], and suggested that CDT induces the activation of the DSB-related pathway. To better characterize the CdtB intracellular effects, the activation and the recruitment of different DDR proteins to damaged sites have been studied, demonstrating that CDT exposure recapitulates the different steps of DSB signaling (Figure 3). First, the three subunits of the MRN complex have been shown to form nuclear foci following CDT exposure [67,68,69]. Different studies highlighted an activation of the ATM-dependent pathway, since the ATM protein level was increased and its phosphorylated form accumulated after CDT infection [11,53,70]. Besides, γH2AX foci are formed in response to CDT from various origins [53,67,68,71,72,73,74,75]. γH2AX is the most commonly used DSB biomarker, but can be observed in response to other stresses, such as replicative stress, hypoxia, chromatin remodeling, senescence or cell death [76]. Hence, to strengthen the fact that CDT induces DSB, another DSB marker, 53BP1, has been shown to form foci after CDT treatment [53,73,75]. Finally, ATM activation in response to CDT leads to CHK2 phosphorylation [11,53,70,72,73]. CHK2 phosphorylates different effectors, such as the cell cycle regulators of the phosphatase family, CDC25A and CDC25C. It is well known that ATM and CHK2 activate and stabilize p53, enhancing the transcription of the p21 gene involved in the cell cycle arrest at G1/S [77]. As expected, several studies showed that CDT induces p53 phosphorylation and stabilization, leading to the accumulation of p21 [38,73,78].

Few publications studied the activation of the ATR-CHK1 pathway in response to CDT. In 2006, Taieb *et al.* showed the phosphorylation of ATM, CHK2 and CHK1 proteins following a 24-h treatment with EcCDT. However, in this study, no information on the protein responsible for the CHK1 phosphorylation was given [72]. In a recent publication, the activation of the ATM-CHK2 and ATR-CHK1 pathways, in response to gamma-irradiation or CDT treatment, were compared [53]. Irradiated GM637 fibroblasts present a rapid and transient ATM-dependent CHK1 phosphorylation that precedes a second wave of CHK1 phosphorylation, mediated by ATR, which was not activated during the first 8 h post-irradiation. This delayed ATR-CHK1 pathway activation, as well as the late CHK2 phosphorylation are a consequence of the unrepaired DNA lesions that stall and ultimately collapse the RFs when cells progress through the S-phase. In contrast, following HdCDT treatment, the kinetics of ATR and CHK1 phosphorylation are totally different, while the ATM-CHK2 response is quite similar. Indeed, ATR is found phosphorylated at early time points after CDT exposure, with the same kinetics as CHK1 phosphorylation, which increases over time and is shown to be ATM-independent [53]. These data show that, in contrast to the IR-related DDR response, the ATR-CHK1 pathway is activated early and continuously in response to HdCDT.

### 2.2. Cellular Effects of the CDT-Induced DNA Damage

#### 2.2.1. Cell Cycle Arrest

The cell-cycle progression is regulated by sequential activation and nuclear relocalization of CDK/Cyclin complexes [79]. At the beginning of the cell cycle, the activation of CDK4/Cyclin D and CDK2/Cyclin E complexes controls the G1-phase progression and entry into S-phase. The CDK2/Cyclin A complex then regulates the S-phase progression. The S-phase completion and G2 transition are coordinated by the activation of the CDK1/Cyclin A complex. Finally mitotic entry depends on the activation and nuclear relocalization of the CDK1/Cyclin B complex. CDK/Cyclin complexes are activated by CDC25-dependent dephosphorylation and can be inhibited by different regulators, like p16, p21, p27 and Wee1 [80].

In response to CDT treatment, CHK2 activation induces the sequestration of CDC25C phosphatase in the cytoplasm, making it unable to activate the CDK1/Cyclin B complex [81]. As a result, the CDK1/Cyclin B complex is hyperphosphorylated [11,52,72,82] and inactive [50], preventing cells from entering the G2-phase (Figure 3). The implication of CDC25C in this process was demonstrated by overexpression of a recombinant CDC25C, which abrogates the CDT-induced G2/M cell cycle arrest [83]. Two other key factors of the cell cycle regulation are p53 and its p21 downstream target. Coherently, the G1/S checkpoint activation in response to CDT relies on the activation of p53 and p21 [84,85,86]. The G1/S checkpoint activation being largely dependent on p53, the variation in the p53 status among the cell types used in the experiments may explain the difference observed for their cell-cycle arrest; most of the p53 negative cells (or with a p53 inactive form) only arresting at G2/M and not at G1/S [68,74,86]. However, p21 induction can also be p53-independent, and a study indeed reported, in response to CDT, a p21 accumulation that was p53-independent [78]. In eukaryotic cells, p21 is known to inhibit many CDK/cyclin complexes, leading to the cell cycle regulation at different steps [87]. CDT intoxication may therefore lead to the cell cycle arrest at the G1/S transition via the CDK2/cyclin E inactivation.

#### 2.2.2. Cell Death or Senescence

Cell cycle arrest is not the only cellular response induced by the DDR. Indeed, the ATM-CHK2 and ATR-CHK1 pathways also activate DNA repair and cell death pathways. Generally, CDT treatment leads to cell death by an apoptotic pathway. CDT-mediated apoptosis has been shown to follow the intrinsic mitochondrial pathway, involving the BAX/Bcl-2 protein [51,74,88], cytochrome C release [74] and caspases activation [74,88,89]. However, few cell lines were shown to use the extrinsic apoptotic pathway through the caspase-8 cleavage [88,89,90]. CDT intoxicated cells die either by apoptosis or necrosis, the latter being perhaps a consequence of abortive mitosis. Hence, some studies demonstrated that in spite of the DDR activation, CDT-treated cells can bypass the G2/M checkpoint, resulting in micronucleation and abortive mitosis [82,91]. Furthermore, endoreplication events have also been documented [49,82]. CDT has been shown to induce an apoptotic response (Figure 3), following the cell cycle arrest, in a broad range of cell lines, with cell death being detectable between two and four days post-intoxication [9]. However, hematopoietic cells seem to activate a rapid apoptotic pathway during the first day of treatment to such an extent that the cell cycle arrest is not even observed [38,51,67,86,92]. When tested under the same conditions, non-hematopoietic cells presented a mild apoptotic response compared to hematopoietic cells, like monocytes and T-cells [93]. Hematopoietic cells therefore show the most dramatic apoptotic response and do not seem to activate the DDR in response to CDT, suggesting a specific cytotoxic mechanism that does not involve CDT-related DNA damage. Hence, such a DNA damage-independent cell death response may rely on the CdtB phosphatase activity [94].

Another possible cellular outcome of the CDT genotoxic effects is the induction of cellular senescence. CDT intoxicated cells express the hallmarks of cellular senescence (*i.e.*, persistently activated DNA damage signaling, enhanced senescence-associated β-galactosidase activity and promyelocytic nuclear compartments expansion) [73]. This is especially important with regard to the senescence-associated secretory phenotype and inflammation [95], as the expression of proinflammatory mediators is observed after CDT infection [18,96]. Finally, this could lead to cytokine-induced bystander genotoxic effects, with a two-fold increase of reactive oxygen species (ROS) observed after a CDT chronic treatment [91]. These particular aspects will not be further outlined in this review.

## 3. Characterization of the CDT-Induced DNA Lesions

### 3.1. CDT Does Not Directly Induce DSBs

Although the well-established model assumes that the CDT genotoxic activity is driven by a direct CdtB-induced DSB formation, several lines of evidence favor a more complex situation. Indeed, if a broad range of studies pointed out the recruitment of many factors involved in DSB signaling and repair as early as two hours after CDT intoxication (see above), DSB formation only occurs 6 h after CDT intoxication and increases over time, as detected by the direct visualization of DSB by pulse field gel electrophoresis [86]. By comparison, using the same technique on irradiated cells, a similar amount of DSBs are detected immediately after cell irradiation. As CdtB reaches the nucleus in approximately 3 h [45], the delay between CdtB nuclear localization and DSB appearance may not be explained by a direct CdtB-related DSB formation.

#### 3.1.1. CdtB Nuclease Activity Primarily Generates SSBs

More strikingly, biochemical characterization of CdtB nuclease activity by the digestion of plasmid DNA clearly demonstrated that CdtB from various origins mostly induces nicks on supercoiled DNA, rather than direct DSB [54,67,97]. CdtB belongs to the endonuclease-phosphatase family, including the mammalian DNase I, as EcCdtB, AaCdtB or HdCdtB share sequence homology and structural similarity with DNase I, notably in their active site and DNA binding motif [22,23,44,54,98]. DNase I has been extensively characterized, and its nuclease activity is well documented. This enzyme is a divalent metal ion-dependent phosphodiesterase that catalyzes the degradation of double-stranded DNA by creating single-strand breaks (SSB), also called nicks, in the presence of magnesium [99]. The addition of calcium strongly stimulates the magnesium-dependent nicking activity, leading to the formation of DSB resulting from the presence of two closed nicks in opposite strands [100]. Such a synergistic effect between magnesium and calcium has also been observed for *Helicobacter hepaticus* CdtB nuclease activity [101]. Moreover, the structural and functional relationship between CdtB and DNase I has also been supported by chimeric AaCdtB-DNase I construct analysis [102]. Altogether, these studies strongly suggest that DNase I and CdtB nuclease activities are very similar. However, several studies have demonstrated that DNase I activity is by far higher than CdtB [67,101]. As DNase I primarily induces SSB, which can be converted to DSB under optimal conditions when two nicks face each other, it is therefore unlikely that CdtB induces direct DSBs.

#### 3.1.2. Different Doses, Different DNA Lesions

**Figure 4:**
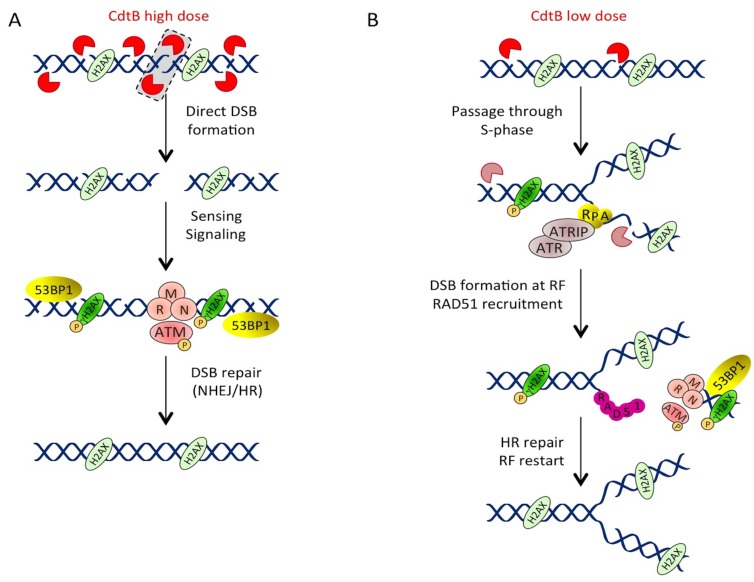
A model of CDT-mediated DSB formation and repair. (**A**) Direct DSB formation with high doses of CDT. CdtB induces a large number of nicks on the host cell DNA. When two closed nicks face each other on opposite strands (dashed rectangle), they directly create a DSB that is sensed by MRN and signaled by ATM, resulting in H2AX phosphorylation and 53BP1 recruitment. This lesion will be repaired by NHEJ or HR. (**B**) Low doses of CDT induce S-phase-associated DSB. At low doses, the number of nicks is not large enough to produce direct DSBs. However, if these nicks are left unrepaired or if CdtB induces nicks during the S-phase (hatched CdtB), these lesions will block RF progression, resulting in ssDNA accumulation. ssDNA is then coated by RPA, leading to ATR activation. Then, the RF collapses, resulting in DSB formation and ATM pathway activation. RAD51 is recruited in order to restart the RF through HR repair.

In light of this rationale, we have been able to show by single-cell gel electrophoresis that EcCDT-treated cells accumulate SSB at early time points of intoxication, before DSB formation [75]. Such an observation may appear contradictory to previous data reporting the signaling of DSB, through the formation of NBS1 or γH2AX foci in the first two hours after CDT treatment [67,69]. This discrepancy is probably due to the CDT doses used in these different studies. Hence, as the vast majority of studies employ doses around 1 µg/mL or more, we chose to evaluate CDT genotoxicity at a very low dose (50 pg/mL) that is yet enough to induce a 50% loss of viability 72 h post-intoxication [75]. It is important to note that increasing doses up to 25 ng/mL induced DSB signaling, via γH2AX and 53BP1 foci formation, as early as 3 h post-intoxication, in agreement with previous reports. Thus, we strongly favor the assumption that CdtB does not directly induce DSBs on genomic or plasmid DNA, but rather SSBs. However, exceeding a certain threshold of SSBs on opposite strands may lead to the generation of DSBs. In a recent study, where the authors worked with high doses of HdCDT (4 µg/mL), the amount of DNA damage detected by a neutral (detection of DSBs) or alkaline (detection of DSBs, SSBs and alkali-labile sites) comet assay was similar after 6 h of exposure to the toxin, leading to the conclusion that HdCDT primarily induced DSBs under these conditions [53]. In contrast, 8 Gy of gamma-irradiation induced a two-fold higher rate of damage revealed under alkaline condition. However, one has to remember that gamma-irradiation induces a large excess of SSBs and alkali-labile sites compared to DSBs, with a ratio of at least 1:20 [103,104]. Besides, the induction of tail DNA under neutral condition in a comet assay not only depends on DSBs, but also on the relaxation of genomic DNA supercoils resulting from SSBs [105], and direct comparison of neutral and alkaline comet assay will therefore under-estimate the SSB:DSB ratio. In conclusion, we believe that cells treated with high doses of CDT rapidly accumulate direct DSBs, these lesions being a part of the total CDT-induced SSBs. At lower doses, the total number of SSBs is not sufficient to produce direct DSBs (Figure 4).

#### 3.1.3. Low Doses of CDT Induce Replication-Associated DSBs

Low doses of CDT thus firstly induce SSBs, which are converted into DSBs during the S-phase [75]. These cells suffer a slight replicative slow-down, underscored by the accumulation of chromatin bound PCNA (Proliferating Cell Nuclear Antigen) -positive cells, which precedes the G2/M arrest. It is therefore tempting to speculate that some unrepaired SSBs are converted into DSBs when encountering an RF (Figure 4), resembling the kinetics of camptothecin-induced DSBs [106]. Camptothecin prevents the repair of the topoisomerase I-induced cleavage complex, causing the RF to collapse and, thus, DSB formation. To corroborate this similarity, we have shown that the camptothecin-induced cell cycle alteration and DSB formation are reminiscent of those induced by low doses of EcCDT [75], suggesting that as for camptothecin, CDT-induced DNA damage requires S-phase progression to convert SSBs into DSBs. However, in contrast to camptothecin, data are missing in order to demonstrate that CdtB-induced SSBs are irreparable. However, CDT-induced DSBs (high doses) are still signaled by the persistence of γH2AX foci two days after a 4-h pulse treatment, whereas the number of γH2AX foci strongly decreases within 4 h after gamma-irradiation [53]. This suggests that the CdtB-related lesions cannot be repaired or that CdtB retains its nuclease activity over a long period of time after intoxication. If the latter is true, replication-associated DSB formed at low doses of CDT should result from newly induced SSBs that encounter an RF during replication, before being repaired.

By activating the DDR, replicative stress is known to lead to cellular senescence [95], providing a barrier to tumorigenesis. DDR downregulation prevents senescence initiation and maintenance, allowing cell proliferation and predisposing cells to oncogenic transformation [107,108]. Interestingly, cells surviving to CDT intoxication rapidly exhibit the hallmark of replicative cellular senescence within the firsts few days, including persistently activated DDR [73]. However, after chronic exposure of several weeks to sublethal doses of CDT, intoxicated cells show impaired DDR activation, bypass the cell cycle checkpoints and do not display the β-galactosidase senescent marker [91]. Thus, the different cellular outcomes of CDT intoxication have to be considered in regards to the toxin dose and the related DDR activation. If the CDT-induced apoptotic response is activated by a huge amount of unrepaired DNA lesions caused by high doses of CDT, then senescence may result from replicative stress induced by low doses of CDT. Finally, cells escaping these responses will eventually undergo oncogenic transformation.

### 3.2. Repair of the CDT-Related DNA Damage

#### 3.2.1. Overview of the Eukaryotic DSB Repair Mechanisms

Eukaryotes have developed two main pathways to repair DSBs, namely non-homologous end joining (NHEJ) and homologous recombination (HR) [109]. NHEJ is the predominant pathway for the repair of DSBs throughout the cell cycle [110], while HR is more dedicated to RF-associated DSBs during the S-phase and G2 [111]. NHEJ is initiated through the recognition of DSB ends by the Ku70/Ku80 heterodimer, enabling the recruitment of the core NHEJ complex composed of DNA-PKcs, XLF and the XRCC4/DNA ligase IV complex (reviewed by [110]). The Ku70/Ku80 complex holds the two ends in close proximity and prevents resection, while the ligation of the two extremities is mediated by the XRCC4/DNA ligase IV complex. On the other side, resection of the DSB-ends by specific nucleases (including MRE11) and helicases results in the formation of ssDNA 3' tails, promoting the HR pathway. The ssDNA 3' tails are coated by RPA, which is subsequently displaced by BRCA2 to allow the formation of the nucleofilament between the ssDNA and RAD51. The nucleofilament can then invade a homologous template, usually the sister chromatid, to initiate the repair (reviewed by [112]).

#### 3.2.2. Involvement of the Direct DSB Response Pathway

Until recently, little was known concerning the pathways involved in the repair of CDT-induced DNA damage. The importance of the ATM-dependent pathway has been clearly demonstrated in the signaling of the CDT-related DSBs, but its role in surviving CDT intoxication is still under debate. ATM-deficient lymphoblastoid cell lines exposed to HdCDT exhibit a reduced p53 stabilization and cytotoxicity, determined by the sub-G1 population, in comparison to wild-type counterparts [66]. In consistency, the apoptotic response of CHK2-depleted keratinocytes treated with AaCDT was attenuated compared to control cells [70]. On the contrary, ATM-deficient fibroblasts are hypersensitive to HdCDT [53]. The difference observed between ATM-deficient lymphoblastoid cell lines, and fibroblasts may be explained by the atypical response of hematopoietic cells to CDT intoxication (see above). In addition, the opposite effects of ATM and CHK2 deficiency were not unexpected, as demonstrated by the radioresistance of *Chk2^−/−^* mice compared to the extreme sensitivity of the *Atm^−/−^* mice to gamma-irradiation [113,114]. Besides, a glioblastoma cell line mutated in DNA-PKcs shows an increased cell death after HdCDT exposure, pointing out that NHEJ is required for the repair of CDT-induced DNA lesions, at least for high doses [53] (Figure 4).

#### 3.2.3. Implication of HR during the CDT-Mediated Replicative Stress Recovery

Beyond the requirement of the ATM pathway to protect cells against CDT genotoxicity, another pathway seems to be activated. Indeed, the pharmacological inhibition of ATM and DNA-PK does not abrogate the phosphorylation of CHK2 after CDT treatment [81], suggesting that ATR may be involved in this response. As a matter of fact, a rapid and increasing activation of the ATR-CHK1 pathway has been observed during the first 24 h post-intoxication with HdCDT [53]. As mentioned above, the ATR activation requires the accumulation of ssDNA regions coated by RPA, and interestingly, RPA foci are rapidly detected in EcCDT-treated cells [75]. One of the first studies characterizing the CDT-induced cell cycle arrest showed that replication seems to play a crucial role in the response to CDT-induced DNA damage, since the progression through the S-phase appears to be essential for cells to be arrested at the next G2/M [50]. The importance of the S-phase is also indicated by the observation that low doses of CDT induce the formation of replication-associated DSBs. As HR plays a major role in the repair of DSBs produced at RF, this pathway is probably activated in response to CDT. The BRCA2 expression level is enhanced in response to HdCDT [53], and RAD51 is recruited at DSBs sites induced with low doses of EcCDT [75]. However, after an 8-h treatment with high doses of HdCDT, 100% of the exposed cells exhibit γH2AX foci, whereas MRE11 foci are only detected in 40% to 45% of these cells [67]. This result suggests that in the context of CDT-induced DNA damage, the mobilization of MRN does not reflect the sensing of all DSBs, but may rather be involved in the DSB processing, probably the resection of S-phase-associated DSBs during HR repair. Interestingly, a yeast genetic screen revealed that only the strains defective for HR, but not for any other repair pathways, including NHEJ, were compromised in surviving to AaCdtB [115]. This study corroborates another yeast screen depicting the CjCdtB sensitivity of a genome-wide mutant library [116]. Besides demonstrating the replicative stress response to CjCdtB and the protective role of HR, this study also pointed out the difference between the repair of HO endonuclease and CjCdtB DNA lesions, suggesting that CdtB-induced genotoxicity is not similar to the direct HO-related DSB. Finally, human RAD51- or BRCA2-depleted cells are hypersensitive to CDT intoxication, confirming the involvement of HR in the repair of CDT-induced DNA lesions in mammals [53,75].

## 4. Conclusions

Chronic exposure to DNA damaging agents may result in genomic instability, because of the alteration of key genes involved in DDR, cell-cycle regulation or DNA repair mechanisms, therefore enhancing the risk of tumor development [117]. Chronic exposure to CDT sublethal doses has been shown to induce genomic instability, with DDR and checkpoint activation being impaired upon genotoxic stress [91]. In addition, the cell viability was not found to be altered, due to sustained p38 MAP kinase activity, and anchorage-independent proliferation, a hallmark of cell transformation, was observed. These results point out the impairment of damage detection and DNA repair mechanisms in chronically CDT-exposed cells. Furthermore, CDT treatment was shown to induce pro-inflammatory genes and was associated with inflammation to dysplasia progression [18]. Inflammation contributes to cancerogenesis [118], and it is known that bacterial pathogens induce host cell DNA damage through chronic inflammation [119]. Bacterial infection has already been linked to carcinogenesis. *Helicobacter pylori* is a well-known example, associated with gastric carcinoma (reviewed in [120]), downregulation of repair mechanisms and increased mutation frequency [121,122], although this pathogen does not encode any CDT genotoxin. New examples are still being uncovered, with recent studies associating *Fusobacterium spp.* with colorectal cancer and providing mechanistic insights into inflammation involvement in this process [123,124]. Now, chronic *S. typhi* infection is also associated with gallbladder and hepatobiliary carcinoma [125]. Altogether, these results suggest that CDT may contribute to virulence and pathogenicity by interfering with various cellular processes, leading to genomic instability and inflammation [96]. A global view is being established, and the clarification of CDT-expressing bacteria involvement in host pathogenicity will be of particular interest in the future. Particularly, in this context, the characterization of CDT genotoxic potential appears essential.
